# Dermatological Disorders following Liver Transplantation: An Update

**DOI:** 10.1155/2019/9780952

**Published:** 2019-04-01

**Authors:** Dipesh Kumar Yadav, Xue li Bai, Tingbo Liang

**Affiliations:** Department of Hepatobiliary and Pancreatic Surgery, The First Affiliated Hospital, Zhejiang University School of Medicine, Hangzhou 310003, China

## Abstract

Patients undergoing liver transplantation (LT) are at a high risk of dermatological complications compared to the general population as a result of long-term use of immunosuppressant. However, the risk is not as high as other solid organ transplantations (SOT), particularly for skin cancer. The liver is considered as an immune privileged organ since it has a low prevalence of humoral rejection in contrast to other SOT, and thus, LT requires a minimal amount of immunosuppressants compared to other SOT recipients. However, because of the large volume of the liver, patients with LT have higher donor lymphocytes that sometimes may trigger graft-versus-host-disease, yet it is rare. On the other hand, the vast majority of the nonspecific dermatological lesions linked with cirrhosis improve after removal of diseased liver or due to the immunosuppressant used after LT. Nevertheless, dermatological infections related to bacteria, viruses, and fungus after LT are not uncommon. Additionally, the incidence of IgE-mediated food allergies develops in 12.2% of LT patients and may present as life-threatening conditions such as urticaria and/or angioedema and hypersensitivity. Moreover, skin malignancies after LT are a matter of concern. Thus, posttransplant dermatological care should be provided to all LT patients for any suspicious dermatological lesions. Our goal is to give an outline of the dermatological manifestation associated with LT for the clinicians by collecting the published data from all archived case reports.

## 1. Introduction

Liver transplantation (LT) possesses a high risk of dermatological complications to the recipient because of long-term use of immunosuppressant drugs after transplantation [1]. However, among all solid organ transplantations (SOT), LT requires a minimal amount of immunosuppressants and sometimes even allows its complete cutoff [2]. Thus, patients who have undergone LT have a lesser tendency of dermatological complications than that of other SOT recipients [3]. Nonetheless, due to the large volume of the liver, LT patients have higher donor lymphocytes compared to other SOT [4]; these donor lymphocytes proliferate owing to the immunosuppression; however, they rarely trigger graft-versus-host-disease [4–6]. Nonetheless, every patient with LT should be provided with posttransplant dermatological care and should be advised for a dermatologist visit for any suspicious dermatological problems [7, 8]. This minireview intends to provide an update on dermatological disorders associated with LT for the clinicians.

## 2. Preexisting Dermatological Conditions after LT

Patients undergoing LT may have preexisting dermatological disorders in association with cirrhosis or chronic liver diseases (Table 1). In most cases, the dermatological disorders improve after removal of diseased liver or due to the immunosuppressant used after LT. The vast majority of the nonspecific dermatological lesions linked with cirrhosis disappear within a few weeks to months following the LT [9, 10]. However, complications like Dupuytren's contractures from cirrhosis are usually irreversible [1]. In spite of the fact that the dermatological manifestations of liver disease such as autoimmune hepatitis, chronic cholestatic liver disease, and hepatitis C after LT have not been well documented, nevertheless studies propose that most of these conditions like alopecia and vitiligo associated with autoimmune hepatitis [11–13], xanthomas and xanthelasmas associated with chronic cholestatic liver disease [14], cryoglobulinemia, porphyria cutanea tarda, leukocytoclastic vasculitis, and lichen planus associated with hepatitis C [1, 15], and Kayser-Fleischer rings (KF rings) associated with Wilson's disease [1] improve significantly; however, KF rings are not considered as dermatological disorder.

Similarly, coexistence of dermatological diseases such as atopic dermatitis and psoriasis may not be the result of liver disease itself. Still, immunosuppressants used after LT may improve the clinical manifestations of such disorders [16]. Nevertheless, rapid tapering in doses of immunosuppressant like Prednisolone may intensify the dermatological disorders.

Likewise, patients undergoing LT may have a past history of treated skin cancer. However, past history of treated skin cancer is not absolute contraindication to LT. Normally, LT should be limited only to those with nonmetastatic nonmelanoma skin cancer and stage I melanoma [17, 18]. Besides, for occult micrometastasis complete radiological evaluation should be done before considering patient for LT. Nonetheless, in case of recurrence, immunosuppressant dose should be decreased; though, it may increase the risk of graft rejection [18].

## 3. Dermatological Complications after LT

As stated earlier, LT possesses a high risk of dermatological complications that includes infections, cosmetic problems, side effects of medications (Table 2), immune-mediated effects from the transplanted liver, and skin cancer as a result of long-term use of immunosuppressant drugs [1, 19, 20].

### 3.1. Infections

Potential etiologies of infection in LT patients are diverse, including bacteria, mycobacteria, viruses, fungi, and/or along with skin coinfection and opportunistic infections of clinical significance only in immunocompromised hosts [1, 21, 22]. Thus, treatment of dermatological infections after LT can be demanding. Commonly occurring bacterial and viral infections are usually seen in the early period after LT, whereas rarer bacteria and opportunistic infections are seen in the late period. After LT, patients are generally in an immunocompromised state, and serologic testing is not generally useful for the diagnosis of infection due to delay in seroconversion [23]. Hence, systematic microbiologic and histologic tests should be performed in the case of doubtful diagnosis. Most of the time, bacterial and viral infections prevail early within three months of LT. However, rarer opportunistic bacterial and fungal infections occur after three months of LT [24, 25]. At the point when a liver transplant recipient presents with an infectious complications, quick response and aggressive treatment are essential for an ideal clinical results. However, the decision of antimicrobial regimens is generally more intricate due to the prerequisite of therapy and the prevalence of drug toxicities and interactions. Nonetheless, resistance to antimicrobial therapy is high among immunocompromised patients [26]. As an antimicrobial generally works in association with the host's immune system to fight infection, immunocompromised patients usually present with dysbiosis in the gut microbiota environment, where the bacterial evolutionary landscape is generally altered, and an immunocompromised hosts are unable to fight against all pathogen replication [26–29]. Indeed, even antibiotic-sensitive strains of pathogen may consequently be able to survive longer with a normal dose of antibiotic in these patients. Further, pathogens strengthen their potential to survive by mutation or horizontal gene transfer [26, 30]. Thus, these considerations must be taken while prescribing any antimicrobial regimens in LT recipients.

#### 3.1.1. Bacterial Infections

Bacteria are the most common cause of infection after LT [31]. Among all the bacterial infections, surgical site infection (SSI) due to Staphylococcus aureus is the most prevalent and accounts to be about 10% of the total bacterial SSI [32]. Other causes of SSI include bacteria, such as E. coli and P. aeruginosa, and fungus like Candida [31, 33, 34]. SSI manifests itself in early period after LT and presents with as redness, swelling, tenderness at the incision site, and purulent discharge from the wound. Risk factors of SSI include prolonged operating time, old age of patients, and a large volume of blood transfusion [32]. Cephalosporins or vancomycin can be used for prevention of bacterial infections and treatment should be done according to susceptibility-guided antimicrobial therapy.

Considering bacterial infections, some potentially life-threatening infections like staphylococcal scalded skin syndrome and necrotizing fasciitis must be taken into account and should be diagnosed and treated as early as possible. Staphylococcal scalded skin syndrome is caused by Staphylococcus aureus and mainly presents with fever and abrupt onset of widespread painful erythroderma and scarlatiniform rash of skin often involving the face, upper extremities, lower extremities, and intertriginous areas [35]. Moreover, within 48 hours, blisters, exfoliation on slight rubbing of the skin (Nikolsky's sign), and extensive desquamation might occur [36]. The diagnosis of staphylococcal scalded skin syndrome is often made clinically. Skin biopsy shows intraepidermal separation, without marked inflammation or necrosis. However, isolation of the organism from blisters, blood, and urine is often negative. Staphylococcal scalded skin syndrome usually can be confused with graft-versus-host disease and toxic epidermal necrolysis. Treatment is generally done with intravenous antibiotics like flucloxacillin or vancomycin [36].

Staphylococcal species can likewise cause necrotizing fasciitis (NF). NF is an overwhelming cutaneous infection, particularly so in the transplant recipient. NF spreads rapidly through superficial fascia, subcutaneous fat, and deep fascia. The most commonly affected areas are the extremities, perineum, and abdominal wall [37]. Nonetheless, NF is associated with a high mortality rate that accounts in between 25 and 30% [37]. Poor immune, old age, diabetes, and alcohol are thought be risk factors for NF, where both male and female are equally affected [37]. Surgical debridement and earlier treatment with broad-spectrum antibiotics addition to intravenous vancomycin or intravenous daptomycin are the mainstay of treatment for NF [37].

Other bacterial infections like cellulitis and necrotizing fasciitis due to E. coli [38, 39], toxic epidermal necrolysis [40], subcutaneous abscesses due to nocardia [41], etc. have been reported in the literature. However, dermatological disorder due to Mycobacterium tuberculosis infection is rare after LT [42]. Skin manifestation of common bacterial infections is enlisted in Table 3.

#### 3.1.2. Viral Infections

Dermatological infections related to viruses after LT are not uncommon (Table 4). A herpes group of viruses is most common among the opportunistic infections caused viruses, of which cytomegalovirus (CMV) is key in terms of its influence on liver transplant outcome [43]. Moreover, it has been found that the rate of infection due to herpes simplex is high as 35%, and reactivation occurs usually within 3 weeks after LT [43, 44]. However, reactivation of latent herpes simplex may also occur after few years of LT that can be associated with severe hepatitis [45, 46]. Dermatological manifestations associated with herpes simplex are generally atypical and present with necrosis, torpid ulceration, and pseudotumoral mass [1]. On the other hand, reactivation of herpes zoster virus infection that presented as necrotic or hemorrhagic pustules, was generalized in distribution, and was limited to dermatome was reported to affect less than 5% of liver graft recipients [47]. Recognition of these skin lesions and treatment with proper antiviral drugs like acyclovir or valacyclovir on time may be lifesaving. If untreated, it can trigger unexpected life-threatening complications.

Likewise, CMV infection occurs in cases of strong immunosuppression and presents with polymorph vesicles, ulceration, necrotic lesions in oral cavity, and genitalia [43, 48]. Additionally, reactivation of human herpes viruses 6 and 7 in immunocompromised recipients within 2 to 8 weeks of LT has also been found [49, 50]. Patients usually present with fever, cutaneous rash, myelosuppression, encephalopathy, and rejection [49, 51]. Besides, clinical manifestations like verrucae vulgaris, condyloma, and plantar warts associated with Human papilloma viruses [1], lingual infection associated with Epstein Barr-virus [52], and erythema infectiosum and vasculitis associated with Parvovirus B19 (PVB19) infection [53] have also been reported in LT patients.

#### 3.1.3. Fungal Infections

Fungal infections after LT have obtained significant considerations as a result of their relationship with high morbidity and mortality. Despite the fact that different fungal species infection has been identified in LT recipients, the most common infections are due to Candida species [54] followed by the Aspergillus infection [55]. Cryptococcus neoformans infection is less common, which usually presents as meningitis, lungs infection, and cellulitis [56]. Moreover, endemic mycoses because of Histoplasma capsulatum, Coccidioides immitis, and Blastomyces dermatitidis may develop in LT patients from endemic areas, and among these, Coccidioides immitis generally remains and needs prolonged treatment [57]. Furthermore, infection with Alternaria species, Sporothrix schenckii, and Trichophyton rubrum is less common; however, these have been reported to cause invasive infection in liver transplant recipients [58]. Treatment of fungal infection after LT is often done with broad-spectrum antifungal agents such as caspofungin, micafungin, and anidulafungin [32]. Moreover, combination of antifungal drugs like fluconazole, itraconazole, and amphotericin B is also being used [32]. In fact, the management of fungal infection in transplant patients remains more challenging, because of drug-drug interactions between azole antifungals (e.g., fluconazole, itraconazole, and voriconazole) and immunosuppressants drugs impact the concentrations of immunosuppressant and puts the patient at increased risk for either drug toxicity or even graft rejection [59–62]. Subsequently, it is difficult undertaking for the clinician to keep up immunosuppressant concentrations within the appropriate therapeutic range.

### 3.2. Skin Cancer

Skin malignancies in liver transplant patients have been a matter of concern (Table 5). The incidence of skin cancer is strongly associated with the use of immunosuppressant drugs after LT [63], exposures to sunlight [64], and infection with papilloma viruses [65]. The most commonly occurring skin cancer is squamous cell carcinoma followed by basal cell carcinoma [66]. Moreover, it has also been found that the risk of developing melanoma increases more frequently by 2.5 to 4 times after LT [63]. Similarly, the risk of developing Kaposi's sarcoma increases by 400 to 500 times after LT. Kaposi's sarcoma has been found to be strongly associated with Human herpesvirus-8 (HHV-8) infection, where 95% of Kaposi's sarcoma lesions harbor HHV-8 infection [67]. Nonetheless, cases of Merkel cell carcinoma after LT have also been reported [68]. In one study, 18% of patients presented with actinic keratoses, which is a preneoplastic lesions and a precursor of SCC. Moreover the study found that the risk of developing actinic keratoses was associated with older patients, the use of cyclosporine, and the patients having skin phototypes II and III. Additionally, this study also suggested that LT patients with prior treated skin cancer should undergo follow-up every 3 months and all other LT patients should undergo follow-up yearly for complete skin examination [21].

### 3.3. IgE-Mediated Food Allergies

In recent years, there have been a number of cases reporting on the development of IgE-mediated food allergies after LT [69, 70]. The incidence of IgE-mediated food allergies is reported to be developed in 12.2% of LT patients and estimated to be 3 times higher in the first year of the transplant [70]. Moreover, these IgE-mediated food allergies may present as life-threatening conditions, for example, urticaria and/or angioedema and hypersensitivity [70]. However, the pathogenesis behind food allergies after liver transplant is not well understood; tacrolimus is believed to contribute in the development of food allergies [71].

### 3.4. Graft-versus-Host Disease (GVHD)

Graft-versus-host-disease (GVHD) is a rare complication after LT, with a frequency of 0.1–2% [72]. In a recent study, the median time for onset of GVHD was 28 days. Moreover, GVHD presented with skin rash (92%), pancytopenia (78%), and diarrhea (65%) in the LT patients. Additionally, it was also frequently associated with Enterobacter bacteremia, invasive Aspergillosis, and disseminated Candida infections [73]. GVHD usually involves the skin, mucosa, liver, and the gastrointestinal tract. However, it also targets other organs, including the immune system. Skin manifestations of GVHD typically begin with characteristic blanchable erythematous macules rash on the ears, palms, and the soles [6, 74]. Additionally, it also may involve the neck, cheek, and back portion in the earlier period [6, 74]. Nevertheless, in the late periods acute cutaneous GVHD can progress, giving rise to generalized morbilliform lesions with smooth or hyperkeratotic papules and hyperpigmentation [74]. In severe cases of GVHD, patients often develop generalized and intense erythroderma, blisters, or substantial sloughing of skin. Diagnosis of GVHD can be made on clinical evidence; however, skin biopsy can provide definitive diagnosis [74]. Treatment with high potency topical corticosteroids can be done for limited skin involved or mild GVHD [74]. Besides, systemic therapy is usually required in case of severe presentation [6, 74, 75].

### 3.5. Other Dermatological Disorders

As stated earlier, some other dermatological disorders after LT may include cosmetic problems, side effects of medications, and immune-mediated effects from the transplanted liver. Rapid onset of a purpura can be because of a severe thrombocytopenia [76]. About 0.7% of LT patients experiences idiopathic thrombocytopenic purpura (ITP) within a few days to several months [77]. Thrombocytopenic purpura may be due to different causes such as viral infections [78], drug-induced [79], and transmission from the graft liver [80, 81] or associated with an underlying immune liver diseases [77, 82]. Furthermore, porokeratosis has also been reported in some literatures after LT [83, 84], which is an indication of strong immunosuppression, and typically occurs 4 to 5 years after transplantation [84].

## 4. Prevention and Management

Prevention of dermatological complications following LT focuses primarily on skin cancer prevention. After LT, patients should protect themselves from sunlight with sun protective clothing and sunscreens with a sun protective factor (SPF) of 30 or higher [85, 86]. In addition, a patient's skin should be examined completely and systematically, including the oral, genital, and anal areas for any lesions.

## 5. Conclusions

Dermatological disorders after LT have various and in some cases concurrent etiologies. Moreover, they may have a serious course and require close observation. Earliest recognition of cutaneous diseases after LT permits treatment at an early stage, as well as notifying the transplant surgeon that the patient might be excessively immunosuppressed, as shown by the existence of warts, condyloma, porokeratosis, skin cancer, and other dermatological disorders. Every patient with LT should be provided with posttransplant dermatological care and should be advised every 3-month follow-up for the patients with prior treated skin cancer and yearly follow-up for all other LT patients for complete skin examination.

## Figures and Tables

**Table 1:** Preexisting dermatological conditions after liver transplant.

**Preexisting Dermatological Conditions**	**Underlying Disease**	**Conditions After liver transplant**
Atopic dermatitis and Psoriasis	May not be associated with liver disease.	It improves after liver transplant. However, rapid tapering in doses of immunosuppressant like prednisolone may intensify the dermatological disorders.

Livedo reticularis	Primary hyperoxaluria	It improves after liver transplant.

Panniculitis	Alpha-1 antitrypsin deficiency	It improves after liver transplant.

Photosensitivity	Erythropoietic protoporphyria	It improves after liver transplant, but it may take long time.

Vasculitis	Hepatitis C with cryoglobulinemia	It may worsen after transplant as the viremia increases.

Raynaud phenomenon	Primary biliary cholangitis	It improves after liver transplant.

Vitiligo and Alopecia areata	Autoimmune hepatitis	It improves after liver transplant. Alopecia areata improves in the 4 to 12 weeks after liver transplant. However, vitiligo can occur after liver transplant.

Xanthomas and Xanthelasmas	Chronic cholestatic diseases	It improves after liver transplant.

Acanthosis nigricans	Primary biliary cholangitis	It improves after liver transplant.

Dupuytren's contractures	Liver cirrhosis	Irreversible.

Azure lunules	Wilson disease	It disappears after liver transplant.

Cryoglobulinemia, Porphyria cutanea tarda, Leukocytoclastic Vasculitis, and Lichen planus	Hepatitis C virus Primary biliary cholangitis	It improves after liver transplant.

Kayser-Fleischer rings	Wilson's disease	It improves after liver transplant.

**Table 2:** Dermatological complications due to immunosuppressant drugs.

**Immunosuppressant**	**Dermatological complications**
Prednisolone	Acne, allergic dermatitis, cutaneous and subcutaneous atrophy, dry scalp, edema, facial erythema, hyper or hypo-pigmentation, impaired wound healing, increased sweating, petechiae and ecchymoses, rash, sterile abscess, striae, suppressed reactions to skin tests, thin fragile skin, thinning scalp hair, urticaria.

Mycophenolate mofetil	Acne, edema of the face and extremities, erosive stomatitis, immune thrombocytopenia, flushed, dry skin, mofetil hypersudation, sweating.

Tacrolimus	Gingival hyperplasia, immune thrombocytopenia

Azathioprine	Acne, bleeding gums, angioedema, isolated localized exanthem, erosive stomatitis, skin rashes, alopecia, acute febrile neutrophilic dermatosis.

Cyclosporine	Anaphylactic reactions, edema of the face and extremities, epidermal cysts, folliculitis, gingival hyperplasia, hypertrichosis, onychopathy, sebaceous hyperplasia, urticaria.

Sirolimus	Exfoliative dermatitis, angioedema, rash, acne, impairment of wound, healing, hypersensitivity vasculitis, burning eyes, dry eyes, excessive tearing, itchy eyes, capillary leak syndrome, immune thrombocytopenia, stomatitis.

Everolimus	Peripheral edema, redness, warmth, swelling, oozing, or slow healing of a wound or surgical incision, easy bruising, cold hands and feet.

Cyclophosphamide	Temporary alopecia, delayed wound healing, changes in skin color, severe skin reaction (swelling of face or tongue, burning of eyes, skin pain, followed by a red or purple skin rash that spreads (especially in the face or upper body) and causes blistering and peeling), changes in nails.

Muromonab-CD3	Anaphylactic Reactions- angioedema (including laryngeal, pharyngeal, or facial edema), rash, urticaria, and pruritus.

Rituximab (used in ABO-incompatible liver transplantation)	Urticaria, itch, rash, stomatitis, alopecia, flushing, dry skin, pale skin, bleeding gums, pinpoint red spots on the skin, and sweating.

**Table 3:** Bacterial skin infection after liver transplant.

**Bacteria's**	**Skin Manifestations**	**Treatment**
Mycobacterium tuberculosis	Mycobacterium tuberculosis infection is rare after transplantation.	Directly observed Therapy with following drugs: pyrazinamide, rifampin, ethambutol, and isoniazid.

Nocardia	Responsible for subcutaneous abscesses and nocardiosis.	For lymphocutaneous type combination therapy with imipenem and cefotaxime, amikacin and TMP-SMX. However, superficial skin infections often resolve with empiric antibiotics.

Staphylococcus aureus	Responsible for surgical site infection, pyoderma, staphylococcal scalded skin syndrome, and toxic shock syndrome.	Intravenous flucloxacillin

E. coli	Responsible for necrotizing fasciitis.	Surgical debridement and earlier treatment with broad-spectrum antibiotics in addition to intravenous vancomycin or intravenous daptomycin.

Streptococcus	Responsible for impetigo contagiosa, cellulitis, and ecthyma.	Topical mupirocin antibiotic ointment or retapamulin ointment for 5-7 days.

Bartonella	Responsible for bacillary angiomatosis.	Erythromycin appears to be the antibiotic of choice and is given until lesions resolve, usually within 3-4 weeks of starting therapy. Other antibiotics used include doxycycline, TMP-SMX, tetracycline, and rifampicin.

Pseudomonas aeruginosa	Responsible for ecthyma gangrenosum and necrotizing fasciitis.	Surgical debridement and earlier treatment with broad-spectrum antibiotics in addition to intravenous vancomycin or intravenous daptomycin.

Nontuberculous mycobacteria (Mycobacterium marinum, M. haemophilum, M. fortuitum, M. chelonae, M. abscessus, and M. ulcerans, or M. immunogenum)	Responsible for macular erythema, nonhealing ulcers, erythematous nodules, and papules.	The treatment regimens vary greatly depending on the species and treatment may be required for at least 12 months.

TMP-SMX: trimethoprim-sulfamethoxazole.

**Table 4:** Viral skin infection after liver transplant.

**Viruses**	**Skin Manifestations**	**Treatment**
Herpes simplex	It has been found that the rate of infection due to herpes simplex is high as 35 %, and reactivation occurs usually within 3 weeks after liver transplantation. Dermatological manifestations associated with herpes simplex are generally atypical and present with necrosis, torpid ulceration, and pseudotumoral mass.	Antiviral medications including acyclovir, famciclovir, and valacyclovir for up to a year, with reassessment at the end of therapy.

Cytomegalovirus	Less common, but one of the most important infectious complications after liver transplantation. It usually occurs in the setup of strong immunosuppression. Skin manifestations include polymorph vesicles, ulceration, necrotic lesions in oral cavity, and genitalia.	Antiviral medications such as ganciclovir (GCV), valganciclovir (VGCV), foscarnet (FOS), and cidofovir (CDV) for 3-6 months.

Herpes zoster	Herpes zoster virus infection that presented as necrotic or hemorrhagic pustules, is generalized in distribution, and is limited to dermatome. It affects less than 5% of liver graft recipients.	The nucleoside analogues acyclovir, valacyclovir, or famciclovir can be used for 7 days.

Epstein-Barr virus	Responsible for lingual infection.	Acyclovir, desciclovir, ganciclovir, interferon-alfa, interferon-gamma, adenine arabinoside, and phosphonoacetic acid.

Parvovirus B19	It presents with erythema infectiosum and vasculitis.	Intravenous immunoglobulin (IVIG)

Papillomavirus	Clinical manifestations like verrucae vulgaris, condyloma, and plantar warts.	Antiviral medications such as podophyllotoxin, trichloroacetic acid (TCA), bichloroacetic acid (BCA), and interferons. Sometimes cryoablation, surgical excision, and laser ablation are also used.

Human Herpes virus 6 and 7	Reactivation usually occurs in the setup of strong immunosuppression within 2 to 8 weeks of liver transplantation and presents with cutaneous maculopapular rashes.	HHV-6 infections in immunocompetent patients are generally not treated, since most cases are self-limited and antiviral therapy has not been studied in such patients. In severe cases of HHV-6 ganciclovir can be used. No treatment is available for HHV-7 infection at present. In vitro, foscarnet, cidofovir, and tenofovir inhibit HHV-7 replication by achievable concentrations. The virus is relatively resistant to acyclovir, penciclovir, and ganciclovir.

**Table 5:** Skin malignancies in liver transplant patients.

**Skin malignancies**	**Remarks**
Squamous cell carcinoma (SCC)	Most commonly occurring skin cancer in liver transplant patients. The cumulative incidence of developing SCC is 32 % after 1 year, 59 % after 3 years, and 72 % after 5 years, respectively, after LT.

Basal cell carcinoma (BCC)	Second most commonly occurring skin cancer in liver transplant patients. The cumulative incidence of developing BCC is 32 % after 1 year, 49 % after 3 years, and 51 % after 5 years, respectively, after LT.

Melanomas	The risk of developing melanoma increases by 2.5 to 4 times more frequently after liver transplantation.

Merkel's cell tumors (MCC)	The incidence of MCC is rare; it commonly occurs in sun-exposed sites. It can be aggressive with rapid growth, local recurrence, lymph node invasion, and distant metastases.

Kaposi's sarcoma (KS)	The risk of developing KS after liver transplantation increases by 400 to 500 times and accounts for 14 % of all malignancies in liver transplant patients.

Cutaneous lymphomas	Cutaneous lymphomas are rare after liver transplantation.
